# Development and Evaluation of a Low-Drift Inertial Sensor-Based System for Analysis of Alpine Skiing Performance

**DOI:** 10.3390/s21072480

**Published:** 2021-04-02

**Authors:** Isidoro Ruiz-García, Ismael Navarro-Marchal, Javier Ocaña-Wilhelmi, Alberto J. Palma, Pablo J. Gómez-López, Miguel A. Carvajal

**Affiliations:** 1 ECSens, iMUDS, Department of Electronics and Computer Technology, ETSIIT, University of Granada, 18014 Granada, Spain; isirg@ugr.es (I.R.-G.); ajpalma@ugr.es (A.J.P.); 2 SkiingLab, iMUDS, Department of Physical and Sport Education, Sport and Health University Research Institute (iMUDS), University of Granada, 18007 Granada, Spain; inv.ismaelnm96@ugr.es (I.N.-M.); fjocana@ugr.es (J.O.-W.); pjgomez@ugr.es (P.J.G.-L.); 3 Human Lab, iMUDS-Sport and Health University Research Institute (iMUDS), Department of Physical Education and Sport, Faculty of Sport Sciences, University of Granada, 18007 Granada, Spain

**Keywords:** inertial sensor, accelerometer, kinematics, alpine skiing, kinetics, photogrammetry 3D

## Abstract

In skiing it is important to know how the skier accelerates and inclines the skis during the turn to avoid injuries and improve technique. The purpose of this pilot study with three participants was to develop and evaluate a compact, wireless, and low-cost system for detecting the inclination and acceleration of skis in the field based on inertial measurement units (IMU). To that end, a commercial IMU board was placed on each ski behind the skier boot. With the use of an attitude and heading reference system algorithm included in the sensor board, the orientation and attitude data of the skis were obtained (roll, pitch, and yaw) by IMU sensor data fusion. Results demonstrate that the proposed IMU-based system can provide reliable low-drifted data up to 11 min of continuous usage in the worst case. Inertial angle data from the IMU-based system were compared with the data collected by a video-based 3D-kinematic reference system to evaluate its operation in terms of data correlation and system performance. Correlation coefficients between 0.889 (roll) and 0.991 (yaw) were obtained. Mean biases from −1.13° (roll) to 0.44° (yaw) and 95% limits of agreements from 2.87° (yaw) to 6.27° (roll) were calculated for the 1-min trials. Although low mean biases were achieved, some limitations arose in the system precision for pitch and roll estimations that could be due to the low sampling rate allowed by the sensor data fusion algorithm and the initial zeroing of the gyroscope.

## 1. Introduction

Alpine skiing is a sport in which turning technique plays a major role in sporting performance. Thanks to new technologies, it is possible to have precise information about what happens during the execution of the turn. Information about acceleration, angles and times is essential to evaluate each turn as well as the overall performance of the skiers. Thus, Spörri et al. showed the characteristics of a world-class giant slalom athlete [1].

Different systems and methodologies have been proposed to monitor skiers’ performances over the years, as well as technical execution. Among them, we highlight those carried out with video analysis for turn detection [2]. A 3D video-based method was also implemented to examine kinematic, kinetic, and energetic variables related to skier technique during slalom race simulations [3]. Moreover, a high kinematic accuracy global navigation satellite system (GNSS) was evaluated to detect the technique of classic cross-country skiing [4]. In addition, Stöggl et al. studied the analysis of the type, number, and duration of the cross-country skiing cycle based on head movement and net vertical displacement [5].

Wireless portable inertial sensors, so-called inertial measurement units (IMUs), are currently employed for monitoring sport activity [5]. Furthermore, this technology can be used for injury prevention and skiing performance enhancement [6]. Camomilla et al. [7] described the different uses of inertial sensors in sport, which are motor capacity assessment, technique analysis, activity classification, and physical demands assessment. Martinez et al.’s study focused on the development and validation of a methodology for the accurate detection of turns in the field as well as identifying different types of runs [8]. Moreover, Neuwirth et al. classified the different types of skiing styles, parallel (drifted or carved) and non-parallel (snowplow or snowplow-steering) turns, based on a GNSS and IMUs [9]. Furthermore, Snyder et al. validate a wearable system for edge angle estimation during simulated alpine skiing on a ski ergometer. That system provided an accurate estimation of the edge angle at long and short turn durations, as well as various slope inclinations [10].

This technology alone or combined with others previously mentioned has also been applied to alpine skiing, simplifying the data gathering and improving the skier comfort during tests [11,12,13]. Data from these systems along with the snow conditions, skier speed, and slope inclination can provide valuable information for improving skiing technique and minimizing injuries. In this line, Martínez et al. developed an automatic algorithm to detect skiers’ turns in an ergometer, with an IMU attached to each ski boot. Their proposed system was able to determine the turn switch point with a precision of ±0.03 s using one or two IMUs mounted to the cuff of the boot [13]. Depending on the variables or factors to analyze or evaluate, the location of the inertial sensor is very relevant. Yu et al. analyzed different locations for an IMU on the skier’s body, concluding that the pelvis was the best location for the only participant in the study [12]. All of these works provided remarkable insights into the monitoring of skiing performance. However, these studies present some limitations for use in actual ski training due to, for example, the very limited capture volume, small number of turns, heavy data post-processing, complex sensor setup [12], and testing in conditions much different from real ones [13]. The aim of the current work is to design and evaluate a reliable, comfortable, and low-cost wireless system based on IMU for analysis of alpine skiing performance.

## 2. Materials and Methods

**Figure 1:**
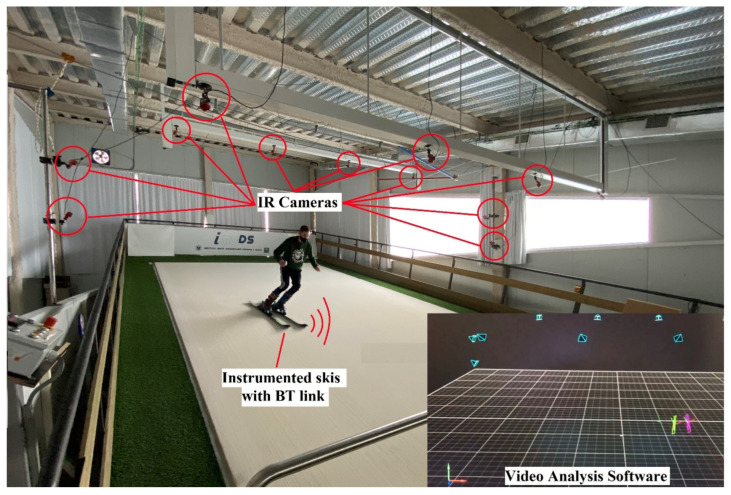
A skier during an experimental test in the ski simulator located at the Sport and Health University Research Institute (iMUDS), University of Granada, Granada, Spain. Infrared (IR) cameras, instrumented skis with Bluetooth (BT) link, and a screenshot of the video analysis software are shown.

Figure 1 shows a comprehensive scheme of the full experimental setup for the IMU system evaluation based on a 3D photogrammetric system with infrared (IR) cameras as the gold standard. Our proposed wireless system consisted of instrumented skis, each including an IMU, pre-processing electronics, support for better video recording, and rechargeable power supply. Alpine skiing runs were carried out in a ski simulator. Tests were carried out in a MaxxTracks ISB2000 ski simulator (© MaxxTracks Indoor Skislopes, Beverwijk, The Netherland) designed for training, research, and teaching purposes, the dimensions of which are 12.50 × 6.55 m (48 m^2^) (see Figure 1). Its surface speed can be varied between 1 and 21 km/h, and the slope ranges from 10° to 19°. Surface wetting is required for achieving suitable skier sliding. This ski simulator is located at the Sport and Health University Research Institute (iMUDS), University of Granada, Granada, Spain.

For evaluation purposes, a 3D photogrammetric system (© 2021 NaturalPoint, Inc. DBA OptiTrack, Beaverton, OR, USA) was used as the gold standard. It is composed of a ring of 12 Flex 3 (resolution: 640 × 480, frame rate: 100 FPS) infrared (IR) cameras oriented to the ski slope. The camera 3D system requires infrared markers attached to the skis to detect their positions. The usually employed passive markers cannot be used on the skis, because their reflective surface would be shielded by the wet surface splashing, preventing good sight of the ski’s position. To overcome this limitation, as is explained below, active IR markers with light-emitting diodes (LED) were included in the skis. Data from the cameras were processed by the optical motion capture software Motive (© 2021 NaturalPoint, Inc. DBA OptiTrack, Beaverton, OR, USA). This software analyses the camera recordings and stores times, positions, and inertial angles (pitch, yaw, and roll) of each ski. Signals obtained by the camera system are interpolated and smoothed by a cubic spline and a low-pass fourth-order Butterworth filter (with a default cutoff frequency of 6 Hz), and the position uncertainty to less than 0.2 mm is calculated by the system software.

### 2.1. Wireless Portable System Design

**Figure 2:**
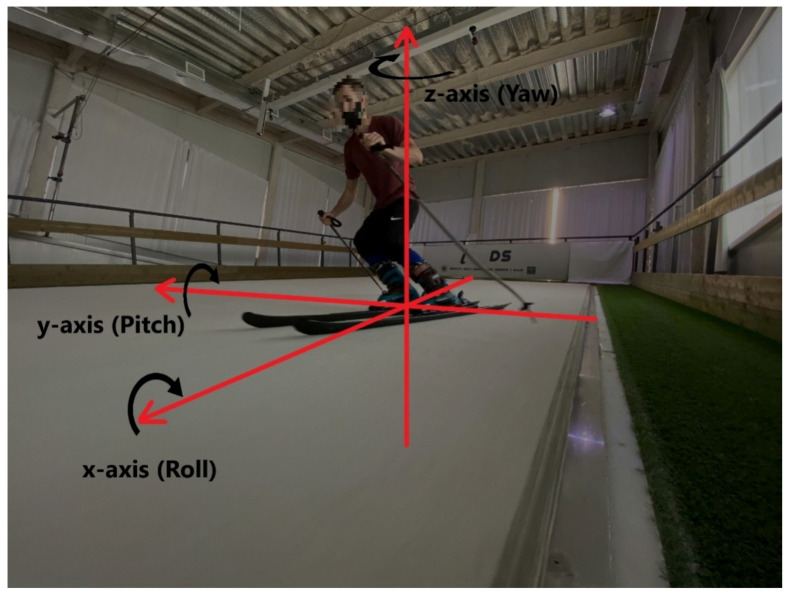
Rotational axes of the ski turn.

The 9DoF Razor IMU board (SparkFun Electronics, Niwot, CO, USA) was selected, which is intended to obtain information attitudes and three-dimension orientation, or the so-called attitude heading reference system (AHRS). This low-cost electronic board combines an MPU-9250 (Invensense, San Jose, CA, USA) 9 degree-of-freedom (3-dimensional accelerometer, gyroscope, and magnetometer) IMU, and an onboard SAMD21 low-power microcontroller (Microchip Technologies Inc., Chandler, AZ, USA) with digital motion processor (DMP) technology that can perform filter processing and an accurate calculation AHRS to determine the ski orientation, namely the inertial angles of yaw, roll, and pitch. Table 1 shows IMU sensor range configurations, accuracies, and cutoff frequencies of the digitally-programmable low-pass filter, f_c_. Sensor settings were selected because these fit with the measurement range, allowing maximum resolution. Moreover, this board was supplied with a micro-SD memory card slot, allowing for IMU data logging. The firmware provided by the IMU manufacturer was reprogrammed to synchronize the IMU and the camera system, setting a sampling rate of 100 Hz (maximum attainable including DMP features). Skier orientation, inertial angles, and coordinate axes (x, y, and z) are shown in Figure 2.

Just after powering up, an initial accelerometer and gyroscope calibration procedure was undertaken consisting of an IMU standing on a levelled flat surface for 10 s. The gyroscope was adjusted to 0°/s in all axes, and the accelerometer was set to 1 g in the z axis and zeroed in the rest of the axes. No user assistance was required to start it.

As mentioned above, to allow for correct skis positioning by the 3D camara system, five 830 nm IR LEDs VSMG2700 (Vishay Intertechnology, Malvern, PA, USA) were soldered on a PCB (printed circuit board) and placed on the front and rear part of each ski. Four LEDs (positioning LEDs) were used to monitor the ski movement, and the fifth one (sync LED) was used to set the starting of the test, as detailed below. The spectrum of the IR LEDs was measured in order to ensure that the maximum frequency emission was tuned with the maximum frequency response of the IR cameras. The photo spectrometer HR2000+ (Halma, Amersham, UK) was used to acquire the emission curve of the LED, obtaining a maximum emission wavelength of 830 nm. Other parameter used to select the LEDs was the emission angle, which was set as wide as possible. VSMG2700 LED presents an emission angle of ±60°, which was wide enough for our application. Despite the 12 cameras and the active markers, some data can be lost due to the dead angles. Therefore, Motive software integrates all the camera data, and in the case of a maximum of ten missed data points, interpolates and filters the adjacent data to provide a smoothed signal.

**Figure 3:**
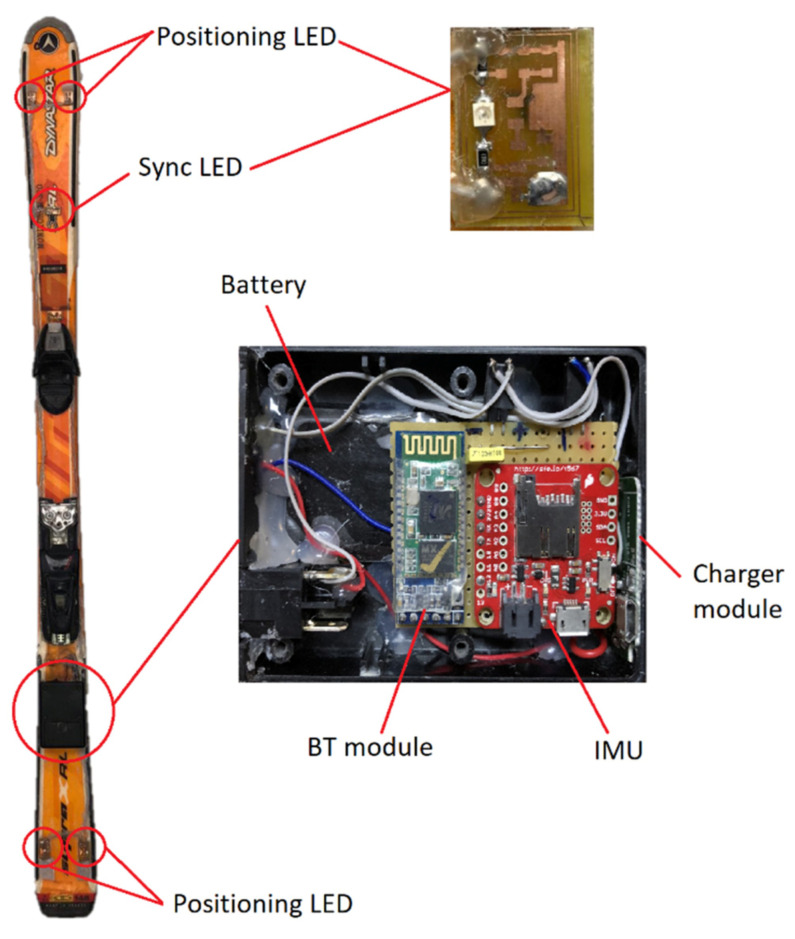
Developed system showing a photo of the ski with the 4-positioning light-emitting diodes (LEDs), an LED for synchronization, and the small waterproof box including the inertial measurement unit (IMU) board, the Bluetooth (BT) module, and the rechargeable battery.

Regarding hardware powering, a RS PRO high-capacity rechargeable battery (Electrocomponents, London, UK) was selected. It was disassembled and placed in a 1593QBK (dimensions 112 mm × 66 mm × 28 mm) waterproof box (Hammond Manufacturing Ltd., Guelph, ON, Canada) that was firmly attached to the ski, behind the boot, including the IMU and processing and communication electronics as well, as shown in Figure 3. The power bank had a 3.7 Ah lithium-ion rechargeable battery. For wireless linking, a HC-05 programmable Bluetooth (BT) module (Guangzhou HC information technology Co., Ltd., Guangzhou, China) was used to send data from the IMU to the computer. Each IR LED consumed 50 mA, the IMU board consumed about 20 mA, and the Bluetooth module, 50 mA. Therefore, the system had an autonomy of more than 10 h.

### 2.2. Test Protocol

Three male expert skiers (age = 33 ± 8, height = 174 ± 7 cm, and weight = 70 ± 8 kg) participated in the study. Participants were ski teachers and coaches with the Spanish Winter Sports Federation (SWSF) with more than 10 years of experience. All participants were monitored with the same device to avoid system reproducibility error. The measurement process involved the following steps:

1. 3D camera calibration. Prior to the recording, it was necessary to calibrate the system to set the space and the ground plane where the activity took place, according to the Motive software documentation [14].
2. IMU zeroing: The skier stood on a flat platform with a slope of 0 degrees for 10 s in order to establish the zero level for gyroscope and accelerometer calibration. In the next section, the importance of this step is pointed out, showing data drift with and without it.
3. Synchronization marks: To synchronize the IMU and the 3D camera system, the skier jumped at the beginning and end of the test, as was done in Martinez et al. [8]. In addition, to check that the mark generated by the sync LED was correct, the sync LED turned off when the IMU-based system began to collect data, providing a visible benchmark in the 3D camera recording.
4. The participant began to ski while the cameras collected the position and inertial angles of both skis, and the IMUs sent inertial angle and 3D acceleration data to the computer. To perform periodic turns, the start of each turn was marked with a metronome. The skier listened to the metronome signal through a Bluetooth headset. The cadence was set at 40 turns per minute and 1.5 s per turn cycle (0.66 Hz) based on the average duration of giant slalom turns [15]. As mentioned, skiers made continuous turns with a speed of 20 km/h and a slope of 12°. Two different trials (three replicas of each one) were performed in these conditions:
  a. 10-min tests for studying the IMU-based system drift. In this study, the full trial duration was analyzed.
  b. 5-min tests for system assessment. In this case, 40 turns (1 min) and 120 turns (3 min) in the central part of each test were considered for the analysis. The central part of test was chosen to skip initial (warm-up) and final (fatigued skier) periods.

### 2.3. Data Analysis

First, to perform the data analysis, inertial angles obtained by the proposed system and by the video system had to be compared. To do that, video system data needed to be denormalized and filtered:

1. The information from the cameras was denormalized to be able to compare and relate with the IMU’s inertial angles. This was necessary because the 3D photogrammetric system data was given normalized between −1 and 1, and the inertial angles provided by the IMU were obtained between 0 and 360 degrees.
2. To avoid comparing between invalid data, samples showing a deviation greater than 20% of the inertial angle ranges (see Table 2) were replaced by an average of ten previous and ten subsequent samples (twenty in total).
3. Then, the moving average of twenty samples was calculated.

Pearson’s correlation coefficient was used to check the correlation between the inertial angles from the video system and the IMU board [16]. The concordance correlation coefficient (Lin CCC) was also calculated [17]. Whereas the ordinary correlation coefficient (Pearson’s) is immune to whether the biased or unbiased version for estimation of the variance is used, the concordance correlation coefficient is not. Schober et al.’s approach for correlation coefficient interpretation was considered. Terms such as very strong correlation and strong correlation were applied for 0.9–1.0 and 0.70–0.89 correlation coefficient values, respectively [18]. Moreover, Bland–Altman analysis (BAA) parameters, including mean bias, standard deviation, and 95% limits of agreement (LoA) [19], and root mean square error (RMSE) were calculated to provide information about the magnitude of error for IMU-based system evaluation. The accuracy and precision of the system was defined as the mean of the error and the standard deviation of the error, respectively, between the IMU-based system and the 3D photogrammetric system [10]. Accounting for the lack of agreement in the revised literature, no qualitative terms (such as acceptable/good/excellent) were used for the interpretation of BAA parameters or RMSE.

To complete the information about the skier’s performance, the acceleration module in the xy plane was obtained from the IMU accelerometer. These acceleration data can also be considered as complementary to those given by the 3D photogrammetric system, where accelerations were not available. The moving average of twenty samples was also applied to smooth these data. The acceleration of the z direction was not relevant in this study.

## 3. Results

In this section, the impact of the IMU zeroing procedure in the drift data to determine the reliability of the proposed system as a function of the test duration was checked. Moreover, the correlation, accuracy, and precision of the inertial angles estimated by the IMU-based system compared to the video system were shown. After system evaluation, inertial angles and acceleration data were combined to assess preliminarily the skiing technique.

Table 2 shows the measured angular ranges and time drifts of inertial angles without and with the application of the IMU zeroing procedure. Ten minute tests showed clear differences among parameter drifts. A sharp reduction of time drift could be observed when the IMU zeroing procedure was carried out, with a reduction factor between 0.02 (yaw) and 0.19 (roll). Assuming an absolute angular drift of 2° as a tolerable limit, according to a previous work [20], our IMU-based system could provide a strong correlation during 11 min in the worst case (roll). Consequently, the IMU zeroing procedure was thereafter always applied in the rest of the performed test.

**Figure 4:**
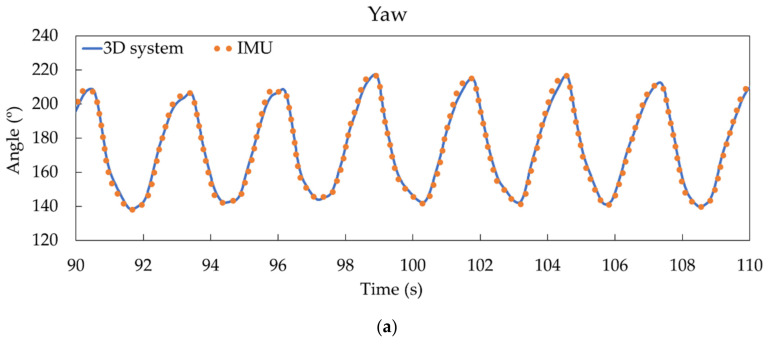

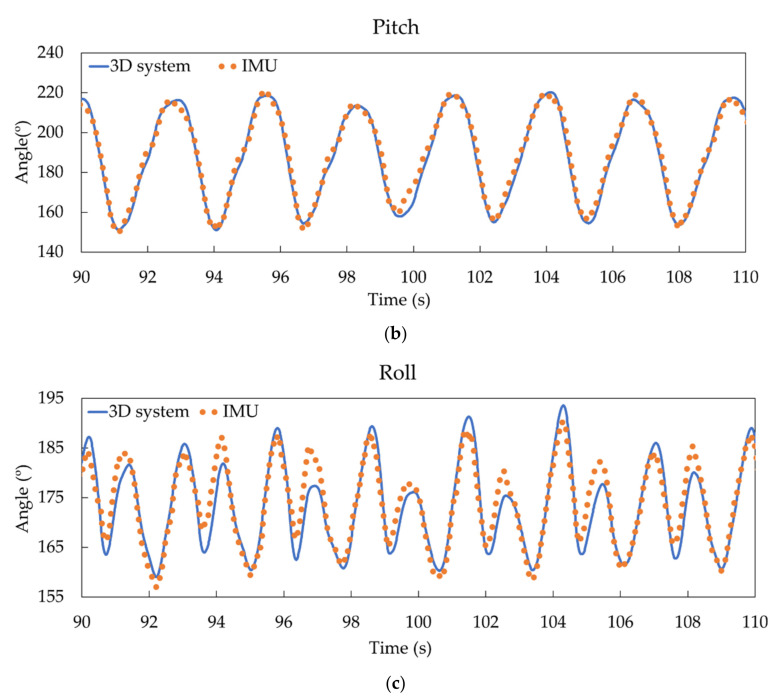
Comparison of IMU-based system and video system data for participant 1. Yaw angle (**a**), pitch angle (**b**), and roll angle (**c**).

Regarding the system evaluation with the 5-min trials, some results of direct comparisons can be observed in Figure 4 for the inertial angles for participant 1.

To quantify the comparisons depicted in Figure 4, different mean correlation coefficients were calculated for the 40-turn test, as shown in Table 3. Correlations around 0.89, 0.95, and 0.99 were obtained for roll, pitch, and yaw, respectively, with Lin CCC and Pearson coefficients. The RMSE between systems showed worse values for pitch and roll than yaw and for 3-min than for 1-min analyzed periods, ranging from 1.47° (yaw) to 5.24° (pitch). Similar trends were obtained for system precision (standard deviation (SD) and LoA) from 1.46° to 4.65° for yaw and roll, respectively. High accuracy (with angle means measured by both systems below 1.2°) was obtained, showing improvement for even longer tests.

**Figure 5:**
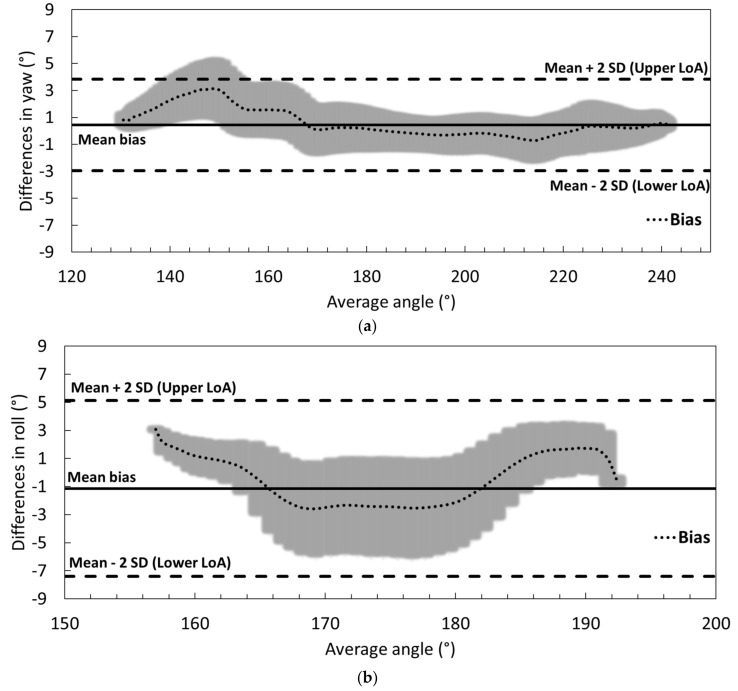

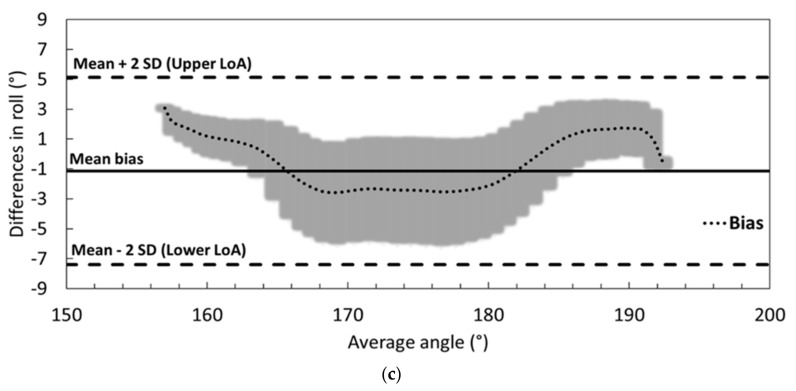
Differences between systems for (**a**) yaw, (**b**) pitch, and (**c**) roll for the 40-turn study for the three participants’ aggregated data (1-min study). Dotted lines shows biases and shaded areas their standard deviations as a function of the average angle. Positive and negative values indicate underestimation and overestimation of the IMU-based system, respectively. Mean biases and LoA (Mean + 2·SD) from Table 3 are also represented as solid and dashed straight lines, respectively.

Figure 5 displays the Bland–Altman plots of differences in yaw, pitch, and roll between the reference and the IMU-based systems for the 1-min study for the aggregated data of the three participants. Solid and dashed straight lines show the mean biases and mean LoA of each inertial angle, respectively (Table 3). Dotted lines represent the biases as a function of angle, with shaded areas displaying their standard deviations. In the yaw plot, a clear angle underestimation (positive differences) can be observed in the 140° to 170° angular interval. However, lower biases and differences were measured compared to the other two angles. From Figure 5b, lower differences were observed in the initial and final part of the turn (higher and lower angles) than in the central angular interval. With a similar general trend, higher differences were observed in the central angular zone between 165° to 185° in roll (Figure 5c). In Figure 4, this fact can also be seen with higher differences in this angular interval. In short, there is a tendency of angular overestimation (negative differences), around −3° in average, in the central angular interval, with an opposite trend (positive differences) in the low and high angular intervals for pitch and roll.

**Figure 6:**
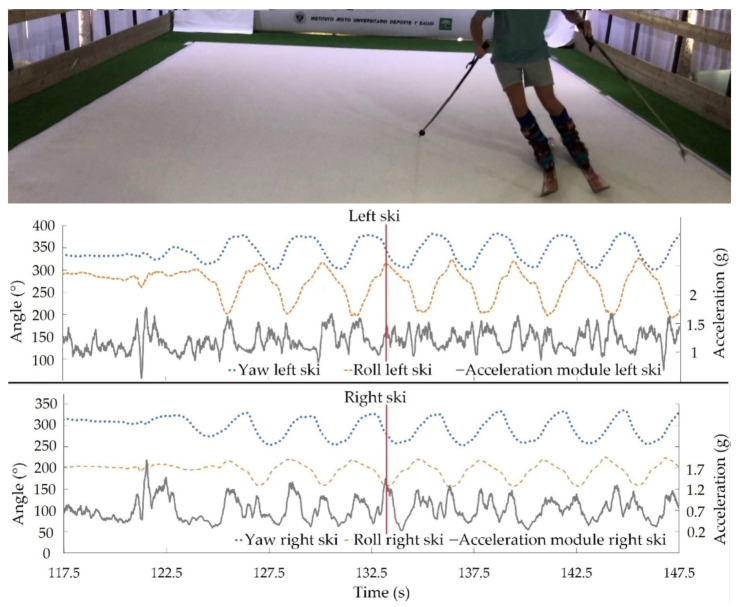
Synchronized yaw, roll, and acceleration modulus in the xy plane for each ski around the beginning of a turn. Vertical line shows the point of maximum xy acceleration of turns by participant 1.

**Figure 7:**
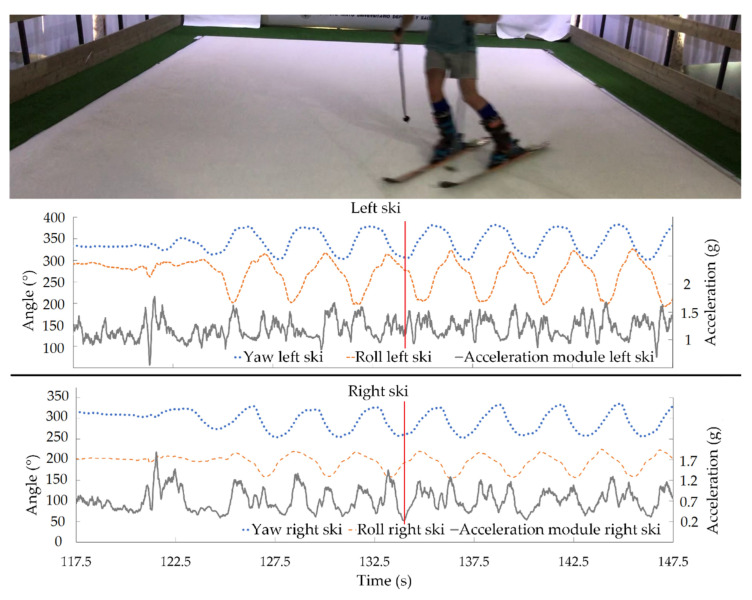
Synchronized yaw, roll, and acceleration modulus in the xy plane for each ski around the end of a turn. Vertical line shows the point of minimum xy acceleration of turns by participant 1.

A skier’s polished turn technique is essential for correct skiing performance. Therefore, aiming at shedding light on this issue, the acceleration data were added to the inertial angles to assess turning technique, highlighting its kinematic characteristics. The lateral angle of the ski and the acceleration of the ski determine how each turn is made. These data can provide relevant information about when and at what angle the skier can speed up. Figure 6 and Figure 7 show synchronized data obtained by our designed IMU-based system around the beginning and end of a turn, respectively. Results represented are the most relevant inertial angles, yaw and roll, and the acceleration modulus in the xy plane for left and right skis. Vertical lines point out the exact times corresponding to the photos. Maximum and minimum xy accelerations are marked in Figure 5 and Figure 6, respectively.

## 4. Discussion

The aim of this pilot study was to develop and verify the feasibility of the designed IMU-based system for monitoring the turn technique in alpine skiing. One of the priorities of our design was the simplicity both of the design and deployment of this evaluation system. Let us remember that the IR LEDs were placed for improved detection of the gold standard system and for synchronization purposes. Consequently, those optoelectronic components would not be included in the final system, which only comprises the waterproof box, with the electronic board inside, attached to each ski, and the software for processing and presentation of data. Hardware dimensions can be downsized to 50 mm × 40 mm × 10 mm with a minimum weight of 15 g without the battery. The battery can be adapted to the required test duration; therefore, a lighter and smaller battery could be used instead of the one here mentioned. Although hardware was located in the ski’s rear area behind the boot to reduce water splashing, it can be placed at any other part of the ski.

Another advantage of wireless IMU-based systems is that they not are affected by data loss due to blind spots of any camera system. This can happen when IR marks (passives or actives) are shielded by the skier’s body movements or when they get wet. Therefore, with IMU-based systems, data loss is minimized. Furthermore, the selected electronic board has a slot to insert a micro-SD memory card for continuous data logging. This reduces the information loss caused by a Bluetooth link failure.

The results of Table 2 demonstrate that assuming an absolute angular drift of 2° as a tolerable limit [19], the IMU-based system can provide low-drifted data for more than 11 min of continuous usage in the worst case (roll). If only the most relevant inertial angles for this kind of movement are considered (yaw and pitch), this duration can be extended to 13 min. This is possible due to the IMU zeroing procedure that can be conducted prior to the test. Moreover, this zeroing procedure is automatically performed when powering up the system by simply remaining still on a flat surface for 10 s.

Regarding the measured inertial angles, according to Table 3, the better correlation of yaw and pitch can be explained due to their higher angular ranges compared to roll. Given the kinematic characteristics of the movement under study, a skiing turn, angular variations are larger in the xy plane, corresponding to yaw and pitch, than in the z direction (roll). Therefore, the shorter the range, the more affected by uncertainty sources in both systems, resulting in a lower, although strong, correlation [18]. As a result, the correlations of the angle data obtained by our IMU system implemented on the skis are similar to those obtained in the studies by Martinez et al. (95.3–99.7% comparing IMU system with 2D video) [8] and Neuwirth et al. (89–95% comparing IMU system with GNSS) [9], regardless of the fact that in these studies, the IMU was placed in the ski boot.

On the other hand, the RMSE and BAA parameters showed that although a high accuracy (low mean bias) can be achieved with our system even for long lasting trials, it provided low precision for pitch and roll estimations. This low precision was presented mainly in the central angular intervals of the turn when IMU was not able to accurately track them (see Figure 5b,c). Biases in different angular intervals showed opposite signs, providing lower mean biases but higher standard deviations in both 1- and 3-min analyzed periods. Issues such as differences in data processing, IMU inaccuracy, and/or IMU low sampling rate could explain this behavior. In the used IMU, DMP processing (responsible for the sensor data fusion and data drift compensation) limited the sampling rate to 100 Hz. This fact could explain the low precision in some angle estimations. For example, it has been noted that a 200 Hz sampling data rate is required for accurate monitoring of running activity [21]. In this sense, a faster IMU will be included in future wearable systems in line with a recent reported system [22].

Data from Figure 6 and Figure 7 can be used to inform both the athlete and the coach how the acceleration varies in each turn, as well as the ski’s edge angle. Thanks to this information, corrective feedback can be generated to help improve the skier’s performance on each descent, enabling them to perform the descent in a shorter time and with optimal technical execution. Moreover, the ski edge angle at each phase of the turn to generate speed is also depicted. In the case of the image and taking into account the direction of the skis at the start of the turn (Figure 6), the moment of maximum acceleration is reached when the skis are facing the fall-line, with the smallest ski edge angle. On the other hand, when the skis are crossing the fall-line at the end of the turn (Figure 7), the acceleration of the skis decreases to a minimum and the ski edge angle is greater. This means that when the athlete is moving away from the line of maximum slope the pressure increases on the ski with greater edge engagement. That results in an increase of the frictional force and, therefore, in a lower acceleration and speed. The application of force on the skies implies acceleration variations, and therefore speed changes in turns.

As an additional improvement compared to previous systems, our system provides reliable results for longer periods of time with reduced drift (Figure 4). In fact, magnetometer data allows the system to correct the gyroscope’s drift. The longest test that was carried out was 650 s, observing a drift of 1.43° in the roll angle (the worst case). The speed of the simulator was set at 20 km/h, which means that a descent of 3600 m was monitored. The tests in Figure 4, Figure 5, Figure 6 and Figure 7 had a duration of 300 s at a speed of 20 km/h, corresponding to a descent of about 1400 m. Furthermore, accuracy and precision were only evaluated for this speed; all these features would allow the system to be transferred to real tests in snow without making changes. On the track, a complete system validation study with more participants and different speeds will be carried out in real conditions.

## 5. Conclusions

A compact, wireless, and IMU-based system was designed and evaluated to study the alpine skiing technique. When compared to the video reference system, very strong correlations for Pearson coefficients [18] were achieved for the yaw and pitch. RMSE and BAA studies showed high accuracy for the three inertial angle estimations, and higher precision for yaw than for pitch and roll estimations. This fact could be attributed to IMU performance limitations in terms of low accuracy and low sampling rate. Additionally, the contribution of acceleration data would allow for an exhaustive analysis of skiing performance and could make it possible for the coach to create a specific training to improve the technique and to correct the skier’s movements. This feedback can be in real time due to wireless connection (Bluetooth link) with a smartphone. Moreover, by completing the IMU zeroing process, which only takes 10 s and does not require participant assistance, long term skiing tests can be undergone up to 11 min with angle drift below 2°. Given the system location, the system comfortability can be assured during skiing, and its features make further research possible.

## Figures and Tables

**Table 1:** Technical parameters of the MPU9250 inertial sensors.

Inertial Sensor (MPU9250)	Selected Range	Sensitivity Scale Factor	Tolerance	ADC (×3)	Low Pass Filer
3D accelerometer	±2 g	16.38 LSB/g	±3%	16 bits	f_c_ = 5 Hz
3D gyroscope	±2000°/s	16.4°/LSB	±3%		
3D magnetometer	±4800 µT	0.6 µT/LSB			

**Table 2:** Experimental angular ranges and time drifts of inertial angles without and with IMU zeroing.

Inertial Angle	Range (°)	Drifts without IMU Zeroing	Drifts with IMU Zeroing
Yaw	130°–240°	6.0 ± 0.1°/min	0.1 ± 0.1°/min
Pitch	150°–220°	1.8 ± 0.2°/min	0.2 ± 0.2°/min
Roll	155°–195°	0.95 ± 0.04°/min	0.2 ± 0.1°/min

**Table 3:** Correlation coefficients, RMSE, and BAA parameters of the inertial angles for the 5-min trials.

	Yaw		Pitch		Roll	
Lin CCC	0.9914		0.9452		0.8892	
Pearson	0.9916		0.9586		0.8901	
Analyzed time (min)	1	3	1	3	1	3
RMSE (°)	1.5	2.5	3.1	5.2	3.4	4.7
Mean bias (°)	0.4	0.4	−0.9	−0.02	−1.1	−0.02
SD (°)	1.5	2.5	3.0	4.5	3.2	4.7
LoA (°)	2.9	4.9	5.8	8.7	6.3	9.1

## Data Availability

The data presented in this study are available on request from the corresponding author. The data are not publicly available due to the amount of data.
